# Exploring low haemoglobin density as a no‐added‐cost screening marker to assess iron deficiency

**DOI:** 10.1111/vox.70135

**Published:** 2025-10-15

**Authors:** Jesse Qiao, Sherif Rezk, Gagan Mathur

**Affiliations:** ^1^ Department of Pathology and Laboratory Medicine University of California, Irvine Orange California USA

**Keywords:** IDA, iron deficiency, iron screening, LHD, low haemoglobin density

## Abstract

**Background and Objectives:**

Iron deficiency (ID), with or without anaemia, affects over 1 billion people globally. Early detection is essential, but current diagnostic tools may be costly, logistically complex and not widely accessible. This study evaluates low haemoglobin density percentage (LHD%), derived from mean corpuscular haemoglobin concentration (MCHC) in routine complete blood counts (CBCs), as a no‐added‐cost, accessible alternative for ID screening.

**Materials and Methods:**

We retrospectively analysed 3526 adult patient records (January–April 2025) from a single‐centre institution, including those with iron panel and CBC performed within 24 h. Patients were categorized as Normal, ID, ID anaemia (IDA) or anaemia without ID (AwoID). LHD% was calculated and assessed across groups using receiver operating characteristic (ROC) curve analyses for cutoff determinations.

**Results:**

Median LHD% increased progressively from the Normal group (2.99%) to the IDA group (6.71%). ROC analysis showed modest but acceptable diagnostic performance: area under the curve (AUC) was 0.677 overall, 0.687 in non‐anaemic and 0.684 in anaemic subjects. Specific LHD% cutoffs varied by context: >4.48% for general screening (61% sensitivity, 67% specificity), >7.02% for donor screening (95% specificity) and >3.74% for anaemia workup (75% sensitivity).

**Conclusion:**

LHD% shows potential as a context‐specific, no‐added‐cost screening tool for ID using existing CBC data. While its diagnostic performance is modest, its scalability and accessibility could support broader public health screening efforts. Further prospective validation is recommended.

**Highlights**

- Low haemoglobin density percentage (LHD%) is a no‐added‐cost, complete blood count–derived marker that shows modest but consistent diagnostic performance for iron deficiency (ID) and ID anaemia screening.
- Context‐specific LHD% cutoffs can optimize sensitivity or specificity depending on clinical needs, including for general population screening, donor evaluation and anaemia triage in hospitalized patients.
- Its scalability and accessibility make LHD% a promising tool for large‐scale screening, especially for resource‐limited settings and public health.

## INTRODUCTION

Iron deficiency (ID), with or without anaemia, remains one of the most prevalent nutritional deficiencies worldwide, affecting over 1 billion individuals [1, 2]. Certain populations are at particularly elevated risk, including surgical patients, pregnant women, individuals with chronic illnesses and repeat blood donors [3, 4, 5, 6]. Early detection is crucial, as untreated ID can lead to fatigue, impaired cognitive and physical performance and decreased quality of life. In patient blood management and transfusion medicine practice, timely identification of ID is critical for guiding appropriate interventions, optimizing blood utilization and improving patient outcomes.

Current laboratory strategies for detecting ID often begin with complete blood counts (CBCs) and iron panel but may be suboptimal for broad ID screening and detection. In addition to missing early detection of ID, additional challenges include panel misinterpretation and lack of general provider awareness of ID [7, 8]. Low serum ferritin and iron saturation (%SAT) are highly specific for ID but lack sensitivity when normal or elevated, especially with concurrent inflammation [7, 8, 9]. Low serum iron levels suggest ID but lack specificity with concurrent inflammation or critical illness and are subject to diurnal variations [10]. Specialized assays, such as the soluble transferrin receptor, zinc protoporphyrin and hepcidin, may show better specificity for ID but may not be readily available across healthcare facilities to facilitate effective clinical decision making [11, 12, 13].

In recent years, reticulocyte haemoglobin (Hb) assays (CHr, Ret‐He) have gained traction as a rapid, yet effective screening tool for the monitoring of ID and have been examined in a variety of clinical scenarios [14, 15, 16, 17, 18]. While reticulocyte Hb–based assays are increasingly recognized in the assessment of iron‐restricted erythropoiesis, their widespread adoption may be limited by access, cost of additional reagents, added technical costs and the need for provider‐driven ordering.

Low haemoglobin density percentage (LHD%), a parameter derivable from the mean corpuscular haemoglobin concentration (MCHC) component of a routine CBC, presents an appealing, yet underexplored alternative [19, 20, 21, 22]. LHD% is non‐proprietary and requires no additional sample preparation—a major advantage as a no‐added‐cost parameter with any haematological analyser's CBC [21, 22, 23]. In contrast to the MCHC [24], LHD% reflects the proportion of hypochromic red cells rather than a mean concentration, an advantage of enhanced sensitivity for detecting early iron‐deficient erythropoiesis.

LHD% in various studies have demonstrated its utility in diagnosing and differentiating ID in several clinical scenarios, including blood‐donor ID screening [19, 20, 21, 22, 25]. LHD% is obtained by exponential transformation of the MCHC using the following equation: LHD% = 100 × √{1 − [1/(1 + e^1.8×(30−MCHC)^)]} [21]. However, LHD%, despite being acknowledged since the early 2000s, has not been thoroughly investigated, relative to ‘traditional’ reticulocyte Hb assay counterparts, for the assessment of iron‐dependent erythropoiesis [17, 26].

Iron‐restricted erythropoiesis first affects haemoglobinization of newly produced erythrocytes and reticulocytes, producing a subpopulation of hypochromic cells even before the MCHC falls. LHD% quantifies the proportion of cells below a Hb density threshold and therefore is more sensitive to early iron‐restricted erythropoiesis than a mean‐based parameter such as MCHC, which can be ‘averaged out’ by coexisting normochromic cells. This population‐level difference explains why LHD% may detect early/functional iron deficits while MCHC remains near‐normal [17, 21, 26].

The aim of this study was to explore the feasibility of LHD% as a screening marker for ID using retrospective, real‐world laboratory data retrieved within a diverse demographic population (tertiary academic medical centre). We aim to evaluate the performance of LHD% as the initial haematological marker for ID detection, which may hold potential for a future universal ID screening tool ideal for resource‐constrained settings and public health research.

## MATERIALS AND METHODS

### Study design and data collection

This is a single‐centre, retrospective initial evaluation study at a tertiary academic medical centre (exempt‐status institutional review board protocol #3441). All unique, consecutive patient records (inpatients and outpatients) from 1 January to 1 April 2025, for patients who had both a CBC and iron panel (serum iron, ferritin, %SAT, total iron binding capacity [TIBC]) performed within the preceding 24 h were extracted using a custom‐built report in the Laboratory Information System (Epic Beaker, Verona, WI, USA). We aim to represent real‐world patient/population distributions with robust sample sizes. All protected health information (PHI) was de‐identified to maintain confidentiality and comply with institutional privacy standards.

### Inclusion/exclusion criteria

Records were retrieved (included) with the first qualifying iron panel and the closest temporally associated CBC collected within 24 h either before or after the iron panel. Repeat or interval test results under the same medical record number were not retrieved. Records were not retrieved (exclusion criteria) for patients younger than age 18. Patients with ≥1 unit(s) of packed red blood cell (RBC) transfusions within the 90 days preceding the iron panel draw date were also excluded in order to mitigate the effects of donor RBCs on patient MCHC, which may bias LHD% calculations. Subjects/records were categorized into mutually exclusive groups: Normal, ID, ID anaemia (IDA), and anaemia without ID (AwoID), based on laboratory criteria (details below). Patient records that did not fall into these four categories were excluded from analysis.

### Group definitions

The ‘Normal’ group included subjects with no abnormalities of the following iron panel and CBC result components: serum iron (Fe), ferritin, %SAT, TIBC, transferrin, RBCs, Hb, haematocrit (Hct), mean corpuscular volume (MCV), mean cell haemoglobin (MCH), MCHC, red cell distribution width (RDW), white blood cell (WBC) count and platelet (PLT) count (our reference ranges are listed in Table S1). The ‘ID’ group included subjects with ferritin <25 ng/mL, or %SAT <20% [8, 27, 28], and normal Hb. The ‘IDA’ group included subjects with ferritin <25 ng/mL or %SAT <20%, with anaemia. Anaemia was classified according to our laboratory reference ranges: Hb <11.5 g/dL for females and <13.5 g/dL for males. The ‘AwoID’ group consisted of subjects with anaemia (Hb below reference range) but no ID (ferritin ≥25 ng/mL and %SAT ≥20%).

**FIGURE 1:**
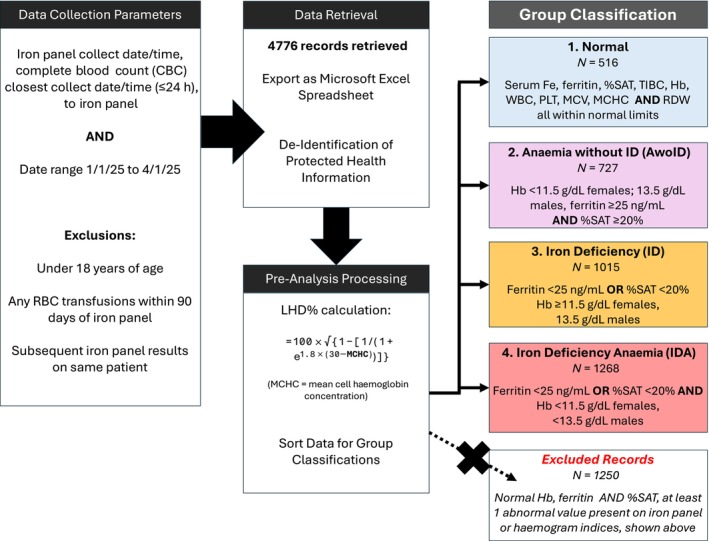
Data retrieval and processing criteria and group classification algorithm. This figure depicts our criteria for data retrieval and processing for group classification. Test abbreviations are provided in the figure. %SAT, iron saturation; Fe, iron; Hb, haemoglobin; LHD%, low haemoglobin density percentage; MCV, mean corpuscular volume; PLT, platelet; RBC, red blood cell; RDW, red cell distribution width; TIBC, total iron binding capacity; WBC, white blood cell.

To ensure a strict and conservative definition of the ‘Normal’ group, all CBC and iron panel parameters were required to fall within reference ranges. Records with any abnormality (e.g., thrombocytopaenia, leukocytosis, macrocytosis or abnormal iron indices not consistent with ID, AwoID or IDA) were excluded from the analysis to avoid potential misclassification in the absence of supporting clinical data. Figure 1 summarizes laboratory data retrieval, processing and group classification criteria.

### Laboratory methods

Iron panels were performed using Beckman Coulter AU680 chemistry analysers (Beckman Coulter Inc., Brea, CA, USA); CBCs were performed using Beckman‐Coulter DxH900 haematology analysers. LHD% was calculated by exponential transformation of the MCHC using the formula: =100 × √{1 − [1/(1 + e^1.8×(30−MCHC)^)]} [21]. Hb thresholds were sex‐specific according to institutional reference ranges and World Health Organization definitions of anaemia. In contrast, ferritin and transferrin saturation (%SAT) thresholds were applied uniformly across sexes, as these are widely accepted biochemical markers of ID and are not typically adjusted for sex in population‐based screening or diagnostic criteria. Our approach allows for independent classification of anaemia and ID, enabling more precise stratification of ID, IDA and AwoID.

### Statistical methods

Statistical analyses were performed using IBM SPSS v29 (Armonk, NY, USA) and plotting LHD% distributions. All *p*‐values <0.05 are considered statistically significant. Continuous variables were summarized as medians [interquartile ranges] because of non‐normal distribution of several parameters. This approach was applied consistently to ensure comparability across groups, regardless of the distribution of each variable. Spearman correlations were performed between LHD% and ferritin, %SAT, and MCV. Receiver operating characteristic (ROC) curve analyses were used to evaluate LHD% diagnostic performance, with area under the curve (AUC) and standard error (SE) reported. To ensure relevance across real‐world diagnostic contexts, we ran ROC analyses on specific groups with cutoff values that were selected based on ROC curve coordinates [29]. Youden's index was used to determine LHD% cutoffs optimized for both sensitivity and specificity. Lastly, we validated our AUC values with a 70/30 random split‐sample approach to assess data consistency.

## RESULTS

Altogether, 3526 records were included in our study (Figure 1), of which 516 were classified into the Normal group, 727 into the AwoID group, 1015 into the ID group and 1268 into the IDA group. Non‐anaemic groups were observed to have lower median ages (Normal: 52 years; ID: 49 years), while both anaemic groups had higher median ages (AwoID: 72 years; IDA: 67 years). Most of the subjects in the ID group were females (77%), while the AwoID group had the lowest number of females (33%). The IDA group had the lowest median Hb (10.2 g/dL), MCV (87.0 fL), MCHC (33.0 g/dL) and %SAT (10%). The ID group had the lowest median ferritin (22 ng/mL). The AwoID group had the highest median MCV (92.4 fL) and ferritin (214 ng/mL), as shown in Table 1. A total of 1250 records without ID or anaemia but had one or more haematological and/or iron panel abnormal value(s) were excluded.

**TABLE 1:** Classification groups and descriptive statistics.

Classification (abbreviation)	*N* (% female)	LHD%	Age, years	Hb, g/dL	MCV, fL	MCHC, g/dL	Ferritin, ng/mL	% iron saturation
Normal CBC and iron panel (Normal)	516 (63%)	2.99 [2.20]	52 [30]	14.0 [1.3]	90.1 [4.8]	33.9 [0.8]	61 [58]	28 [35]
Anaemia without ID (AwoID)	727 (33%)	3.91 [3.85]	72 [23]	10.9 [2.9]	92.4 [9.0]	33.6 [1.1]	214 [505]	30 [17]
ID, without anaemia	1015 (77%)	4.68 [3.72]	49 [32]	13.4 [1.6]	88.1 [7.6]	33.4 [0.9]	22 [43]	16 [9]
IDA	1268 (49%)	6.71 [10.1]	67 [29]	10.2 [2.2]	87.0 [11.3]	33.0 [1.5]	75 [209]	10 [8]
Total	3526 (56%)							

*Note*: Medians [interquartile ranges] are reported for all the above continuous variables.

Abbreviations: CBC, complete blood count; fL, femtolitre; Hb, haemoglobin; ID, iron deficiency; IDA, iron deficiency anaemia; LHD%, low haemoglobin density percentage; MCHC, mean cell haemoglobin concentration; MCV, mean corpuscular volume; *N*, number of subjects in each group.

**FIGURE 2:**
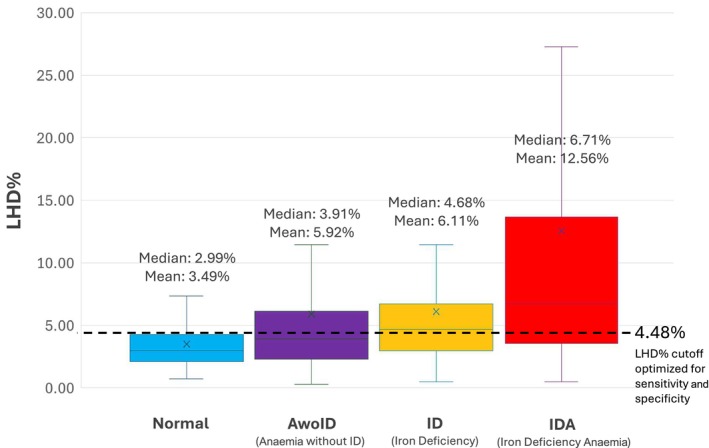
Box‐and‐whisker plots showing distribution of low haemoglobin density percentage (LHD%) within each group classification. This figure depicts box‐and‐whisker plot distributions of the four groups, highlighting non‐normality. Boxes represent the first through third quartiles, while whiskers represent minimums/maximums. Central line represents median, while X represents the mean. Groups are displayed/ordered according to increasing medians. Dotted line represents a representative LHD% cutoff optimized for sensitivity and specificity balance (~60% each) for all study subjects.

Regarding LHD% distributions, the Normal group had the lowest median LHD% (2.99%) with the smallest interquartile range (2.20%), while the IDA group had the highest median (6.71%) with the greatest interquartile range (10.1%). Table 1 summarizes descriptive statistics: age, sex, sample size, LHD% and selected iron/CBC results. Box‐and‐whisker plots (Figure 2) visually depict non‐normal (non‐parametric) distributions of LHD% across all four groups examined, indicated by differences between median (line within box) and mean LHD% (‘x’ within box) values, and unequal quartiles. Across all four groups, the plotted means were consistently greater than the medians.

To rule out whether age and sex are a confounding factor for differences in LHD% among various groups, we performed a binary logistic regression. LHD% was found to be an independent and statistically significant predictor of ID status (Table S2). Weak correlations of LHD% were observed with ferritin, whereas moderate correlations were seen with %SAT and MCV (Table S3).

**FIGURE 3:**
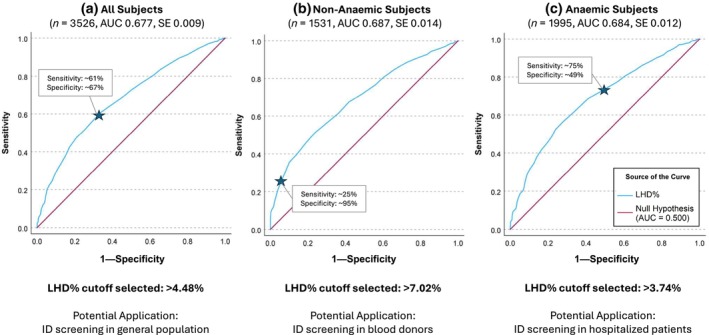
Receiver operating characteristic (ROC) curves for different group classification. All area under the curves (AUCs) show significance (*p* < 0.001) against null hypothesis (AUC = 0.500). Star represents the low haemoglobin density percentage (LHD%) cutoffs suggested. *n*, total number in each analysis; SE, standard error (95% confidence intervals, non‐parametric assumption).

Three separate ROC analyses were done with selected groups of subjects to evaluate LHD% diagnostic performance (Figure 3, Table 2). First, ROC analysis including all subjects (*n* = 3526) yielded an AUC = 0.677 (SE 0.009). The optimized LHD% cutoff (as calculated by the Youden index optimized cutoff for balanced sensitivity and specificity) is >4.48%, with a sensitivity of ~61% and specificity of ~67%. This LHD% cutoff of >4.48% can be used in ID screening within the general patient population.

**TABLE 2:** Receiver operating characteristic analyses and context‐specific low haemoglobin density percentage cutoff for iron deficiency screening.

Groups compared	Clinical context	*N*	AUC	SE	Optimized LHD% cutoff (sensitivity, specificity)	Our selected cutoff (sensitivity, specificity)
All subjects	ID screening in general population	3526	0.677	0.009	>4.48% (61%, 67%)	>4.48% (61%, 67%)
Non‐anaemic subjects	ID screening in blood donors	1531	0.687	0.014	>4.90% (46%, 81%)	>7.02% (25%, 95%)
Anaemic subjects	ID screening in anaemic patients	1995	0.684	0.012	>5.86% (57%, 72%)	>3.74% (75%, 49%)

*Note*: LHD% cutoffs are determined from the coordinates of each selected ROC curve with modest discriminatory power (Figure 3). Selected cutoffs may favour improved sensitivity or specificity by clinical context and may be different from the optimized cutoff (balances sensitivity with specificity, with Youden's index). For example, general patient population screening and anaemia evaluation favour balanced sensitivity and specificity, whereas blood‐donor screening requires prioritizing higher specificity.

Abbreviations: AUC, area under the curve; ID, iron deficiency; LHD%, low haemoglobin density percentage; *N*, total number of subjects for each comparison; ROC, receiver operating characteristic; SE, standard error—each AUC.

Second, ROC analysis including all non‐anaemic subjects with or without ID (*n* = 1531) yielded an AUC = 0.687 (SE 0.014). For this subgroup, Youden's optimized LHD% cutoff is >4.90%, with a sensitivity of ~46% and specificity of ~81%. However, to increase specificity, based on the ROC curve coordinates, we selected an LHD% cutoff of >7.02%, which increased the specificity to 95%, with a corresponding sensitivity of 25%. The aim was to use this cutoff for ID screening in blood donor population.

Third, ROC analysis including all subjects with anaemia with or without ID (*n* = 1995) yielded an AUC = 0.684 (SE 0.012). Youden's optimized LHD% cutoff in this context is >5.86%, with a sensitivity of 57% and specificity of 72%. However, to increase sensitivity, based on the ROC curve coordinates, we selected an LHD% cutoff of >3.74%, which increased the sensitivity to 75% with the corresponding specificity of 49%. The aim was to use this cutoff for ID screening in anaemic patients.

All three ROC analyses demonstrated modest but acceptable discriminatory power of LHD% to effectively screen for ID (AUC 0.677–0.687). When we performed internal validations of our ROC analyses, we found comparable AUC values when we split our data randomly to a 70%/30% mix, versus the AUC values from the entire dataset (Table S4)—suggesting that our ROC models are stable and perform consistently.

## DISCUSSION

Our evaluation suggests LHD% as a potential adjunct, no‐added cost, CBC‐based screening marker for ID in various clinical settings, particularly in resource‐limited and/or large‐scale public health contexts. As a pilot analysis based on retrospective data, our results are intended to inform future prospective studies to further refine diagnostic cutoffs for clinical practice. Compared to earlier LHD% studies [21, 22, 23, 25], we employed significantly larger sample sizes from complex clinical settings to assess real‐world diagnostic performance.

Across diverse patient populations and clinical settings, our study demonstrates LHD%'s ability to identify iron‐restricted erythropoiesis. As shown in Table 1, the median LHD% appears to progressively increase in value as the patient progress from ID to IDA. Advancing age and female sex are known to be associated with higher prevalence of ID [1, 2]; however, we found LHD% to be independent of age and sex. The AwoID group had the highest median MCV (92.4 fL) and ferritin (214 ng/mL) levels, potentially suggesting presence of other aetiologies of anaemia and coexisting inflammatory conditions in this group [30]. Although our dataset did not contain concurrent inflammatory markers (e.g., C‐reactive protein), prior work in inflammatory bowel disease suggests that LHD% may retain diagnostic utility despite concurrent inflammation [19]. Since our study did not differentiate absolute ID versus functional ID (where ferritin is often elevated), we acknowledge the possibility of group misclassification without concurrent inflammatory markers. Because LHD% is a haematology‐derived parameter that reflects the proportion of hypochromic red cells, it may detect iron‐restricted erythropoiesis independent of ferritin decline. This likely explains the weak correlation with ferritin—which is influenced by inflammation—and the moderate correlation with %SAT and MCV.

We performed context‐specific ROC analyses with the aim of examining the utility and ID detection ability of LHD%. The three groups selected with corresponding potential clinical utility were the following: all subjects, for ID screening in general population; non‐anaemic subjects, for ID screening in blood donors; and anaemic subjects, for ID screening in anaemic patients. AUC values across all ROC analyses demonstrated modest but acceptable performance of LHD% as an ID screening biomarker. This is a limitation of our study but also reflects the real‐world challenges that a screening biomarker faces when applied to an expanded and heterogeneous population.

For large‐scale ID screening within the general population, LHD% may offer meaningful impacts on public health, being a no‐added‐cost solution. We included all subjects for this ROC analysis because it included both inpatient and outpatient populations who had iron panels and CBC ordered for various clinical indications, or even as part of routine laboratory evaluations. By balancing sensitivity and specificity, we selected the optimized LHD% cutoff, as suggested by the ROC analysis, for this context. Prevalence of ID in the general US population is 30%–35% [31]. By calculating LHD% from routine CBCs performed during annual physicals and applying a LHD% cutoff of >4.48% (sensitivity ~61%, specificity ~67%), we could proactively and correctly identify ID in ~20 million individuals per 100 million tests performed. Since LHD% cutoffs optimized for sensitivity may generate false positives, we discourage automatic reflex iron panel testing based on an elevated LHD% without clinical context; prudent individual assessment of ID remains critical to minimizing unnecessary subsequent tests.

For ID screening in blood donors, blood donor centres may also consider incorporating LHD% for initial detection. Young donors <23 years of age, pre‐menopausal female donors and frequent donors are at the highest risk for developing ID [4]. The REDS‐II Donor Iron Status Evaluation study suggests that up to 50%–66% of frequent donors may have underlying ID [32]. Blood donor centres have utilized ferritin and other iron vigilance tools that are not logically challenging and costly [4]. In 2021, approximately 6 million volunteers donated blood in the United States [33], out of which 74% were repeat/frequent donors (~4.5 million total). For frequent donor ID screening, specificity should be prioritized to avoid unnecessary follow‐up testing, deferrals and anxiety among healthy individuals. One study adopted a high LHD% cutoff of >9.18% to enhance specificity to reduce false positives, thereby minimizing unnecessary confirmatory iron panel testing and/or donor deferrals [25].

A potential caveat is that our ‘Normal’ cohort was derived from a tertiary academic medical centre and consisted of patients who had iron panels ordered for diagnostic purposes, which we acknowledge may not fully overlap a population of eligible volunteer donors. We therefore consider LHD% >7.02% cutoff to be provisional, requiring validation in donor populations prior to implementation.

From our ROC analysis of non‐anaemic subjects (representing potential blood donor populations), an LHD% cutoff of >4.48% suggested by ROC analysis with a specificity of 77% could lead to a high number of false positives, necessitating unnecessary subsequent follow‐up testing. To achieve a specificity of ~95%, we selected an LHD% cutoff of >7.02% (sensitivity ~25%). Even at 25% sensitivity, if 4 million US repeat donors (assuming ID prevalence 40%) underwent ID screening using the CBC‐derived LHD%, it will accurately defer up to ~400,000 donors with ID each year, thus improving donor safety.

For ID screening in anaemic patients, hospitals and clinics may consider LHD% for rapid detection and triage. Anaemia is quite prevalent in pre‐operative settings and among hospitalized patients [5, 6, 34, 35, 36]. While screening for ID in preoperative anaemia clinics, in hospitalized patients or among patients seeking transfusion alternatives, sensitivity becomes a greater priority. In these scenarios, missing ID diagnoses may lead to suboptimal anaemia management and/or patient outcomes. While RBC transfusions remain useful to treat acute or severe symptomatic anaemia, distinguishing IDA from other causes is essential to guide subsequent therapeutics (e.g., iron supplementation/infusion, erythropoietin, etc.) [36, 37, 38], potentially reducing reliance on allogeneic RBCs and promoting patient blood management best practices.

ROC analysis for this purpose was performed on anaemic subjects; an LHD% cutoff of >5.86% with sensitivity of 57% was suggested by the ROC analysis. However, we selected an LHD% cutoff of >3.74% with a corresponding higher sensitivity of ~75% and specificity of ~49%, thus casting a wider net to capture more cases of ID. Improved patient outcomes from early detection and timely treatment of ID outweigh additional costs that might be associated with follow‐up iron panel testing for false positives. For a tertiary care facility with 30,000 unique annual inpatient admissions, ~40% anaemic prevalence and 30% ID prevalence [5], an LHD% cutoff >3.74% would effectively flag ~2700 true ID cases per year from CBC results, encouraging further ID evaluation.

In conclusion, LHD% offers a compelling, no‐added‐cost screening approach with the versatility and adaptability to support large‐scale ID detection, particularly in community and resource‐limited settings. Our retrospective, uncontrolled analysis of a single tertiary hospital's laboratory records inherently carries selection constraints and potential confounding. To mitigate these, we applied strict laboratory criteria for ID and anaemia classification, adjusted for age and sex, and demonstrated consistent findings through internal validation. Our strict ‘Normal’ group definition improves internal validity but may introduce selection bias, since all non‐anaemic, non‐ID individuals with other haematologic/biochemical abnormalities were excluded. Future prospective studies should validate these thresholds across diverse patient populations, including those with recent transfusions or haemoglobinopathies, and compare LHD% against reticulocyte biomarkers of iron‐dependent erythropoiesis, as reticulocyte Hb biomarkers (e.g., Ret‐He, CHr) are not available at our institution. Such work will further define the role of LHD% in integrated ID screening strategies and enhance its impact on patient care.

While modest in diagnostic performance compared to conventional assays, LHD%'s broad accessibility can facilitate earlier identification of at‐risk individuals and prompt timely confirmatory iron studies. We advocate the use of LHD% not as a replacement for standard iron diagnostics (nor imply any superiority of LHD% vs. existing diagnostic methods), but as an initial flag to guide further clinical assessment of ID. By integrating LHD% screening into routine laboratory workflows, providers can optimize resource utilization and expand ID surveillance without increasing operational burden.

## CONFLICT OF INTEREST STATEMENT

The authors declare no conflicts of interest.

## Supporting information


**Table S1.** Reference ranges used in group selection: Complete blood count and iron panel.
**Table S2.** Binary logistic regression analysis of LHD% predicting iron deficiency, adjusted for gender and age.
**Table S3.** Spearman correlations of LHD% with selected iron deficiency testing biomarkers.
**Table S4.** Internal validation of receiver‐operating characteristic analyses.

## Data Availability

The data that support the findings of this study are available from the corresponding author upon reasonable request.

## References

[1] World Health Organization (WHO) . Worldwide prevalence of anaemia 1993–2005. Geneva: World Health Organization; 2008.

[2] World Health Organization (WHO) . The urgent need to implement patient blood management: policy brief. Geneva: World Health Organization; 2021.

[3] Means RT . Iron deficiency and iron deficiency anemia: implications and impact in pregnancy, fetal development, and early childhood parameters. Nutrients. 2020;12:447. 32053933 10.3390/nu12020447 PMC7071168

[4] Petersen P , Hakimjavadi H , Chamala S , Mathur G . Evaluating utility of routine ferritin testing in blood donors: a hospital‐based blood donor centre experience. Transfus Med. 2024;34:491–498. 39183386 10.1111/tme.13081 PMC11653056

[5] Randi ML , Bertozzi I , Santarossa C , Cosi E , Lucente F , Bogoni G , et al. Prevalence and causes of anemia in hospitalized patients: impact on diseases outcome. J Clin Med. 2020;9:950. 32235484 10.3390/jcm9040950 PMC7230611

[6] Soraci L , de Vincentis A , Aucella F , Fabbietti P , Corsonello A , Arena E , et al. Prevalence, risk factors, and treatment of anemia in hospitalized older patients across geriatric and nephrological settings in Italy. Sci Rep. 2024;14:19721. 39181939 10.1038/s41598-024-70644-8 PMC11344760

[7] Naghii MR , Fouladi AI . Correct assessment of iron depletion and iron deficiency anemia. Nutr Health. 2006;18:133–139. 16859176 10.1177/026010600601800205

[8] Read AJ , Waljee AK , Sussman JB , Singh H , Chen GY , Vijan S , et al. Testing practices, interpretation, and diagnostic evaluation of iron deficiency anemia by US primary care physicians. JAMA Netw Open. 2021;4:e2127827. 34596670 10.1001/jamanetworkopen.2021.27827 PMC8486982

[9] Qiao J , Mathur G . Revisiting a “new” yet “old” approach: reticulocyte hemoglobin and related hematological indices for iron deficiency screening for patient blood management. Transfusion. 2025. 10.1111/trf.18382 41036610

[10] Dale JC , Burritt MF , Zinsmeister AR . Diurnal variation of serum iron, iron‐binding capacity, transferrin saturation, and ferritin levels. Am J Clin Pathol. 2002;117:802–808. 12090432 10.1309/2YT4-CMP3-KYW7-9RK1

[11] Fullenbach C , Stein P , Glaser P , Triphaus C , Lindau S , Choorapoikayil S , et al. Screening for iron deficiency in surgical patients based on noninvasive zinc protoporphyrin measurements. Transfusion. 2020;60:62–72. 31758575 10.1111/trf.15577

[12] Markovic M , Majkic‐Singh N , Ignjatovic S , Singh S . Reticulocyte haemoglobin content vs. soluble transferrin receptor and ferritin index in iron deficiency anaemia accompanied with inflammation. Int J Lab Hematol. 2007;29:341–346. 17824914 10.1111/j.1365-2257.2006.00875.x

[13] Osta V , Caldirola MS , Fernandez M , Marcone MI , Tissera G , Pennesi S , et al. Utility of new mature erythrocyte and reticulocyte indices in screening for iron‐deficiency anemia in a pediatric population. Int J Lab Hematol. 2013;35:400–405. 23176310 10.1111/ijlh.12030

[14] Cheng W , Yanuarso PB , Wahidiyat PA , Rohimi S , Trihono PP , Kadim M , et al. The role of reticulocyte hemoglobin equivalent on the evaluation of iron deficiency and iron deficiency anemia in pediatric cyanotic heart disease: a diagnostic study in Indonesia. BMC Pediatr. 2024;24:541. 39174917 10.1186/s12887-024-05000-w PMC11342583

[15] Choorapoikayil S , Kotlyar MJ , Kawohl L , Pratz PP , Mehic D , Kranke P , et al. Reticulocyte hemoglobin content: a new frontier in iron deficiency diagnostics for major surgical patients. BMC Anesthesiol. 2025;25:40. 39863874 10.1186/s12871-025-02905-6 PMC11762468

[16] Hoenemann C , Ostendorf N , Zarbock A , Doll D , Hagemann O , Zimmermann M , et al. Reticulocyte and erythrocyte hemoglobin parameters for iron deficiency and anemia diagnostics in patient blood management. A narrative review. J Clin Med. 2021;10:4250. 34575361 10.3390/jcm10184250 PMC8470754

[17] Kilic M , Ozpinar A , Serteser M , Kilercik M , Serdar M . The effect of reticulocyte hemoglobin content on the diagnosis of iron deficiency anemia: a meta‐analysis study. J Med Biochem. 2022;41:1–13. 35291499 10.5937/jomb0-31435 PMC8882014

[18] Ogawa C , Tsuchiya K , Maeda K . Reticulocyte hemoglobin content. Clin Chim Acta. 2020;504:138–145. 32014518 10.1016/j.cca.2020.01.032

[19] Farrag K , Ademaj K , Leventi E , Aksan A , Stein J . Diagnostic utility of low hemoglobin density to detect iron deficiency in patients with inflammatory bowel disease. Ann Gastroenterol. 2021;34:521–527. 34276191 10.20524/aog.2021.0622 PMC8276368

[20] Gezgin Yildirim D , Kaya Z , Bakkaloglu SA . Utility of new red cell parameters for distinguishing functional iron deficiency from absolute iron deficiency in children with familial Mediterranean fever. Int J Lab Hematol. 2019;41:293–297. 30624866 10.1111/ijlh.12971

[21] Urrechaga E . The new mature red cell parameter, low haemoglobin density of the Beckman‐Coulter LH750: clinical utility in the diagnosis of iron deficiency. Int J Lab Hematol. 2010;32:e144–e150. 19220525 10.1111/j.1751-553X.2008.01127.x

[22] Urrechaga E , Unceta M , Borque L , Escanero JF . Low hemoglobin density potential marker of iron availability. Int J Lab Hematol. 2012;34:47–51. 21722324 10.1111/j.1751-553X.2011.01355.x

[23] Urrechaga E . Clinical utility of the new Beckman‐Coulter parameter red blood cell size factor in the study of erythropoiesis. Int J Lab Hematol. 2009;31:623–629. 18771498 10.1111/j.1751-553X.2008.01088.x

[24] Schaefer RM , Schaefer L . Hypochromic red blood cells and reticulocytes. Kidney Int Suppl. 1999;69:S44–S48. 10084285 10.1046/j.1523-1755.1999.055suppl.69044.x

[25] Singh A , Chaudhary R , Pandey HC , Sonker A . Identification of iron status of blood donors by using low hemoglobin density and microcytic anemia factor. Asian J Transfus Sci. 2018;12:46–50. 29563675 10.4103/ajts.AJTS_30_17 PMC5850697

[26] Lundgren CR . Implementing reticulocyte hemoglobin into current hematology algorithms. Am J Clin Pathol. 2022;158:574–582. 36048898 10.1093/ajcp/aqac103

[27] Petkova NY , Raynov JI , Petrova DY , Ramsheva ZN , Petrov BA . Diagnostic significance of biomarkers of iron deficiency for anemia in clinical practice. Folia Med. 2019;61:223–230. 10.2478/folmed-2018-0065 31301666

[28] Saboor M , Zehra A , Hamali HA , Mobarki AA . Revisiting iron metabolism, iron homeostasis and iron deficiency anemia. Clin Lab. 2021;67. 10.7754/Clin.Lab.2020.200742 33739032

[29] Habibzadeh F , Habibzadeh P , Yadollahie M . On determining the most appropriate test cut‐off value: the case of tests with continuous results. Biochem Med. 2016;26:297–307. 10.11613/BM.2016.034 PMC5082211 27812299

[30] Weiss G , Ganz T , Goodnough LT . Anemia of inflammation. Blood. 2019;133:40–50. 30401705 10.1182/blood-2018-06-856500 PMC6536698

[31] Tawfik YMK , Billingsley H , Bhatt AS , Aboelsaad I , Al‐Khezi OS , Lutsey PL , et al. Absolute and functional iron deficiency in the US, 2017–2020. JAMA Netw Open. 2024;7:e2433126. 39316402 10.1001/jamanetworkopen.2024.33126 PMC11423176

[32] Brittenham GM . Iron deficiency in whole blood donors. Transfusion. 2011;51:458–461. 21388389 10.1111/j.1537-2995.2011.03062.x PMC3078561

[33] U.S. blood donation statistics and public messaging guide. Available from: https://americasblood.org/wp-content/uploads/2024/01/U.S.-Blood-Donation-Statistics-and-Public-Messaging-Guide-Jan.-2024.pdf. Last accessed 15 Jul 2025.

[34] Barsballe KEB , Bundgaard‐Nielsen M , Ruhnau B , Hillingsoe JG , Aasvang EK , Jans O . Efficacy of a pre‐operative anaemia clinic in patients undergoing elective abdominal cancer surgery. Acta Anaesthesiol Scand. 2024;68:1338–1346. 38986536 10.1111/aas.14495

[35] Klein AA , Chau M , Yeates JA , Collier T , Evans C , Agarwal S , et al. Preoperative intravenous iron before cardiac surgery: a prospective multicentre feasibility study. Br J Anaesth. 2020;124:243–250. 31902590 10.1016/j.bja.2019.11.023

[36] Quarterman C , Shaw M , Hughes S , Wallace V , Agarwal S . Anaemia in cardiac surgery—a retrospective review of a centre's experience with a pre‐operative intravenous iron clinic. Anaesthesia. 2021;76:629–638. 33150612 10.1111/anae.15271

[37] Schaefer B , Meindl E , Wagner S , Tilg H , Zoller H . Intravenous iron supplementation therapy. Mol Aspects Med. 2020;75:100862. 32444112 10.1016/j.mam.2020.100862

[38] Lin Y . Preoperative anemia‐screening clinics. Hematology Am Soc Hematol Educ Program. 2019;2019:570–576. 31808909 10.1182/hematology.2019000061 PMC6913451

