# Supplementary material for: Exploring low haemoglobin density as a no‐added‐cost screening marker to assess iron deficiency
Source: Vox Sang. 2025 Oct 15;121(1):35–42. doi: 10.1111/vox.70135 (PMC12803769; doi:10.1111/vox.70135)
Supplement: Supplementary file 1 — Table S1. Reference ranges used in group selection: Complete blood count and iron panel. Table S2. Binary logistic regression analysis of LHD% predicting iron deficiency, adjusted for gender and age. Table S3. Spearman correlations of LHD% with selected iron deficiency testing biomarkers. Table S4. Internal validation of receiver‐operating characteristic analyses. [file VOX-121-35-s001.docx]

**Supplemental Table 1. Reference ranges used in group selection: Complete Blood Count and Iron Panel**

| **Test Name** | **Reference Ranges** |
| --- | --- |
| White Blood Cell Count (WBC) | 4.0-10.5 x 10^9^/L |
| Red Blood Cells (RBC) | 4.5-5.9 x 10^12^/L (males)  4.0-5.2 x 10^12^/L (females) |
| Hemoglobin (Hb) | 13.5-16.9 g/dL (males)  11.5-15.0 g/dL (females) |
| Hematocrit (Hct) | 41.0-53.0% (males)  36.0-46.0% (females) |
| Mean Corpuscular Volume (MCV) | 81.5-97.0 fL |
| Mean Corpuscular Hemoglobin Concentration (MCHC) | 32.0-35.5 g/dL |
| Red Cell Distribution Width (RDW) | 11.6-14.4 % |
| Platelet Count (PLT) | 150-400 thous/mcL |
| Ferritin | 25-250 ng/mL |
| Iron Saturation (SAT) | 20-55 % |
| Serum Iron (Fe) | 40-180 mcg/dL |
| Total Iron Binding Capacity (TIBC) | 285-500 mcg/dL |
| Transferrin (TRF) | 205-360 mg/dL |

Abbreviations: L, liters; g, grams; fL, femtoliters; %, percentage; ng, nanograms; mcg, micrograms; dL, deciliter

# **Supplemental Table 2: Binary Logistic Regression Analysis of LHD% Predicting Iron Deficiency, Adjusted for Gender and Age**

| **Variable** | **B** | **S.E.** | **Wald** | **df** | **Sig.** | **Exp(B)** | **95% CI for Exp(B)** |
| --- | --- | --- | --- | --- | --- | --- | --- |
| Gender (female vs male) | 0.52 | 0.073 | 50.89 | 1 | <.001 | 1.683 | 1.455 – 1.946 |
| Age (per year) | -0.007 | 0.002 | 16.0 | 1 | <.001 | 0.993 | 0.989 – 0.996 |
| LHD% (per 1 full unit increase) | 0.088 | 0.012 | 53.89 | 1 | <.001 | 1.092 | 1.075 – 1.109 |
| Constant | -2.105 | 0.312 | 45.47 | 1 | <.001 | 0.122 | — |

## Dependent variable: any ID status; independent variables: LHD%, age, and gender. Model Summary: -2 Log Likelihood: 4274.405; Cox & Snell R Square: 0.084; Nagelkerke R Square: 0.116.

**Abbreviations**: B, regression coefficient; S.E., standard error; Wald, Wald chi-square statistic; df, degrees of freedom; Sig., significance (p-value); Exp(B), odds ratio (exponentiated B); 95% CI for Exp(B), 95% confidence interval for the odds ratio; LHD%, low hemoglobin density-percentage

**Supplemental Table 3. Spearman Correlations of LHD% with Selected Iron Deficiency Testing Biomarkers**

| **Correlation** | **Spearman’s Rho [95% CI]** | **P-value** | **Strengths of Association** |
| --- | --- | --- | --- |
| LHD% vs ferritin | -0.192 [-0.225, -0.159] | <0.001 | Weak |
| LHD% vs iron saturation | -0.389 [-0.417, -0.360] | <0.001 | Moderate |
| LHD% vs MCV | -0.412 [-0.440, -0.384] | <0.001 | Moderate |

Strengths of associations in Spearman correlation (Rho) are as follows: weak: 0.10 to 0.39; moderate: 0.40 to 0.69; strong: 0.70 or greater.

**Abbreviations**: LHD%, low hemoglobin density; MCV, mean corpuscular volume; CI, confidence interval.

**Supplemental Table 4. Internal Validation of Receiver-Operating Characteristic Analyses**

| **Groups Examined** | **AUC (SE), Full Data Set (From Table 2)** | **AUC (SE), “Training” Subset (70% of Full Data Set)** | **AUC (SE), “Testing” Subset (30% of Full Data Set)** |
| --- | --- | --- | --- |
| All subjects | 0.677 (0.009)  N = 3,526 | 0.679 (0.011)  N = 2,457 | 0.673 (0.017)  N = 1,069 |
| Non-anemic subjects | 0.687 (0.014)  N = 1,531 | 0.685 (0.016)  N = 1,092 | 0.692 (0.026)  N = 443 |
| Anemic subjects | 0.684 (0.012)  N = 1,995 | 0.689 (0.014)  N = 1,365 | 0.673 (0.022)  N = 626 |

For each ROC analysis performed (Table 2), Internal validation by random split-sample analysis (70/30) demonstrated comparable AUC values across the training (70% of full data set) and testing (30% of full data set) subsets. We observe corresponding standard errors to be marginally increased as expected due to the reduced sample size of the split groups but nonetheless remained within acceptable limits. Across the 70% and 30% split-sample analyses, our observed AUC values remained close to the original full-sample AUCs, with differences ≤0.011 and overlapping standard errors, suggesting stable and robust performance of LHD% in its ability to detect ID.

Abbreviations: AUC, area under curve; SE, standard error; N, total number of subjects in each analysis
